# Validity and reproducibility of a whole‐room indirect calorimeter for measurement of the thermic effect of food

**DOI:** 10.14814/phy2.70740

**Published:** 2026-02-19

**Authors:** Mina Marie Minge, Christine Henriksen, Elin Maria Sandstad Augestad, Rune Blomhoff, Franziska Anna Hägele, Rebecca Dörner, Russell Rising, Stine Marie Ulven, Thomas Olsen

**Affiliations:** ^1^ Department of Nutrition, Institute of Basic Medical Sciences University of Oslo Oslo Norway; ^2^ Department of Clinical Service, Division of Cancer Medicine Oslo University Hospital Oslo Norway; ^3^ Department of Human Nutrition, Institute of Human Nutrition and Food Sciences Kiel University Kiel Germany; ^4^ D & S Consulting Services Inc, Research and Development New York New York USA

**Keywords:** energy expenditure, metabolism, postprandial energy expenditure, thermic effect of food, whole‐room indirect calorimetry

## Abstract

Whole‐room indirect calorimeters (WRICs) measure respiratory exchange, energy expenditure, and macronutrient oxidation. The thermic effect of food (TEF) that occurs after food intake due to digestion, absorption, and storage of nutrients is an important component of daily energy expenditure, but difficult to quantify using metabolic carts. This study evaluated the validity and reproducibility of a 7209 L WRIC for TEF measurement. Technical validation was performed with 7‐h propane combustion tests (*n* = 5), and biological reproducibility was tested in 10 healthy subjects (6 men, mean age 33.5 ± 10.3 years) in replicate 7‐h measurements, after a 3‐day run‐in protocol. Coefficient of variation (CV) and intraclass correlation coefficient (ICC) were calculated for ventilation rates of O_2_ (VO_2_) and CO_2_ (VCO_2_), respiratory exchange ratio (RER; VCO_2_/VO_2_), postprandial energy expenditure, and TEF. Technical validation showed good validity with CVs ranging from 0.23% for RER to 1.74% for energy expenditure. For biological reproducibility, CVs were 3.42%, 1.97%, 2.58%, 3.07%, and 27.48% for VO_2_, VCO_2_, postprandial RER, postprandial energy expenditure, and TEF, respectively. ICCs were excellent for VO_2_ (96%), VCO_2_ (98%), and postprandial energy expenditure (96%), but poor for TEF (24%). In conclusion, the 7209 L WRIC is technically valid and reproducible for ventilation rates and postprandial energy expenditure but showed lower reproducibility for TEF.

## 1 INTRODUCTION

Whole‐room indirect calorimeters (WRICs) can be used to measure different components of thermoneutral 24‐h energy expenditure, which consists of resting (REE) and sleeping energy expenditure, as well as the energetics of physical activity, along with the thermic effect of food (TEF) (Allerton et al., 2021; Dörner et al., 2022; Hall et al., 2019, 2021; Müller et al., 2021; Stinson et al., 2022). TEF is an essential component of 24‐h energy expenditure, referring to the increase in energy expenditure above REE that occurs after the consumption of food due to the processes of digestion, absorption, and storage of nutrients. This contributes between 4% and 10% of 24‐h energy expenditure when individuals are in energy balance (Calcagno et al., 2019; Levine, 2005; Ruddick‐Collins et al., 2022; Westerterp, 2004).

Several factors influence TEF. One key factor is insulin sensitivity, where decreased sensitivity may lead to a reduction in TEF (Camastra et al., 1999). Furthermore, TEF generally declines with advancing age (Du et al., 2014; Jones et al., 2004; Schwartz et al., 1990). In contrast, physical activity tends to enhance TEF (Poehlman et al., 1991). The composition and size of the meal are crucial, as diets with higher energy content typically lead to a higher TEF (Hill et al., 1984; Kinabo & Durnin, 1990; Martin et al., 2000; Nagai et al., 2005; Thyfault et al., 2004). Lastly, both the frequency and timing of meals can affect TEF (Allirot et al., 2013; Tai et al., 1991).

Understanding and accurately measuring TEF is pivotal in the study of metabolism and energy balance. That is why precise measurements of TEF are crucial for researchers and health care professionals, as it provides insight into metabolic health as a possible adjunct for weight management programs (Calcagno et al., 2019). In this context, WRIC emerges as a promising tool for assessing TEF, providing improved accuracy and convenience.

The thermic effect of food is usually reported as the area between the total energy expenditure under inactive conditions and REE curves (Levine, 2005). It is commonly measured between 2 and 6 h postprandially as part of a 24‐h measurement in a WRIC (Kumahara et al., 2011). Reported reproducibility varies, with coefficient of variance (CV) ranging from 21% to 68% (Allerton et al., 2021; Dörner et al., 2022; Piaggi et al., 2013; Ravussin et al., 1986; Tataranni et al., 1995). Studies measuring metabolic rate for 6 h after consumption of moderate to large meals reveal that approximately 57% of TEF is expended at 3 h, 77% at 4 h, and 91% at 5 h, with a peak between 60 and 180 min after a meal (Compher et al., 2006; Reed & Hill, 1996).

Established guidelines for using and reporting from WRIC facilities recommend that validation and reproducibility experiments on energy expenditure and metabolic rates be performed and published (Chen et al., 2020). The aim of this study was therefore to validate a novel WRIC for measurements of TEF. Validation was performed using 7‐h propane combustion tests (technical validation) and replicate 7‐h measurements in healthy men and women to ascertain the reproducibility of ventilation rates of O_2_ (VO_2_), CO_2_ (VCO_2_), the respiratory exchange ratio (RER, VCO_2_/VO_2_), postprandial energy expenditure, and TEF. We also propose that a smaller room can measure TEF more precisely than previous studies.

## 2 MATERIALS AND METHODS

### 2.1 System characteristics, data acquisition, and processing

In this study, the smallest WRIC at the Department of Nutrition, University of Oslo, Norway, was used (Henriksen et al., 2023). The room has an interior volume of 7500 L, determined by the architectural dimensions. After accounting for the volume of the sink, toilet, and the AC unit obtained from the original architect's plans, and the recliner measured with a tape measure, the effective volume of the chamber is determined to be 7209 L. The room has one window with an outside view and one facing the control room. The window facing the control room allows for monitoring of the subject during REE and TEF measurements to ensure protocol adherence.

The Promethion (GA3m2/FG‐250) integrated system (Sable Systems International, Las Vegas, NV) was used to measure respiratory exchange. The system provides minute‐by‐minute measurements of O_2_ consumption and CO_2_ production by measuring the O_2_ and CO_2_ concentrations (%) using high‐grade fuel cells and dual‐wavelength, nondispersive infrared sensors, respectively (Henriksen et al., 2023; Melanson et al., 2010; Rising et al., 2017). Water vapor pressure (WVP, kPa) of the sample gas stream is also measured directly by thin‐film capacitive sensors to correct the ventilation rates (VO_2_ and VCO_2_) continuously (Melanson et al., 2010; Rising et al., 2017). This is accomplished by pulling a fixed amount of fresh air (100 L/min) from the WRIC and collecting a sample from the exhaust side of the system to measure O_2_ and CO_2_ concentrations, as described by Henriksen et al. (2023) and Rising et al. (2017). The flow rate is initially set by Sable System International using a National institute of Standard Technology flow meter, and afterwards minor tweaks are performed as necessary through customizing of the software macros utilizing propane combustion (Rising et al., 2017).

Raw data were collected using Caloscreen 1.3.16 and subsequently processed by ExpeData 1.9.27 (Sable Systems International, Las Vegas, NV), as described earlier (Henriksen et al., 2023; Melanson et al., 2010).

### 2.2 Calibration and equilibration of the instruments

Zero and span calibration of the gas analyzers of the GA3m2/FG‐250 was performed the same day as each propane combustion test, and the day before each measurement of participants. First, nitrogen gas was infused for 25 min and adjusted any CO_2_, O_2_, and WVP readings to zero. Next, a certified span gas with CO_2_ concentration of 0.903% was infused for 5 min, and sensors were adjusted to 0.903% if there were any deviations. The span value for O_2_ is arbitrarily set to a Standard Temperature Pressure Dry (STPD) value of 20.95%. After the zero calibration, an automated equilibration procedure was performed where incurrent air was passed through the desiccant canister to zero the WVP sensors. The air stream was chemically dried using a column desiccant (Drierite, Fisher Scientific). Additional details can be found in Lighton (2019) and Rising et al. (2015, 2017).

### 2.3 Technical validation

For the technical validation, we performed five 7‐h propane combustion tests using instrument grade propane (99.2% purity) (Scott Medical Products, Plumsteadville, PA, USA) in accordance with methods described previously (Rising et al., 2017). Briefly, propane consumed (g) was measured by a calibrated scale (Sartorius Lab Instruments GmBH & Co, Goettingen, Germany), and the weight of the propane was noted at the start and end of the 7‐h measurement for the calculation of the propane burn rate (g/min). The stoichiometry of the complete oxidation of propane for energy expenditure (kcal), VO_2_ (L), VCO_2_ (L), and RER was then compared to what was measured within the WRIC (Rising et al., 2017).

### 2.4 Study participants for reproducibility study

For the biological validation, students and staff from the Department of Nutrition, University of Oslo, were recruited for two measurements in the WRIC, separated by at least 3 days. For female participants of reproductive age not using contraceptives, the measuring days were separated by 4‐week intervals to account for their phase of the menstrual cycle. Recruitment took place between April 2023 and April 2024. Inclusion criteria were age >18 years, body mass index >18 kg/m^2^, and stable weight (±5%) for the past 3 months. Exclusion criteria included medications that affect energy metabolism, smoking, chronic disease, pregnancy, lactation, and self‐reported claustrophobia.

In total, 12 participants were included (7 men and 5 women). Two participants were excluded from the analysis because they did not complete the second measurement day. Therefore, results from 10 participants were analyzed.

Participants signed written informed consents before participating in the study. As the project was considered methodological and not health research, the Regional Ethical Committee provided a letter of exemption (Reference number: 588647). The project was approved by the Data Protection Services (SIKT) (Reference number: 164609).

### 2.5 Study protocol

**FIGURE 1:**
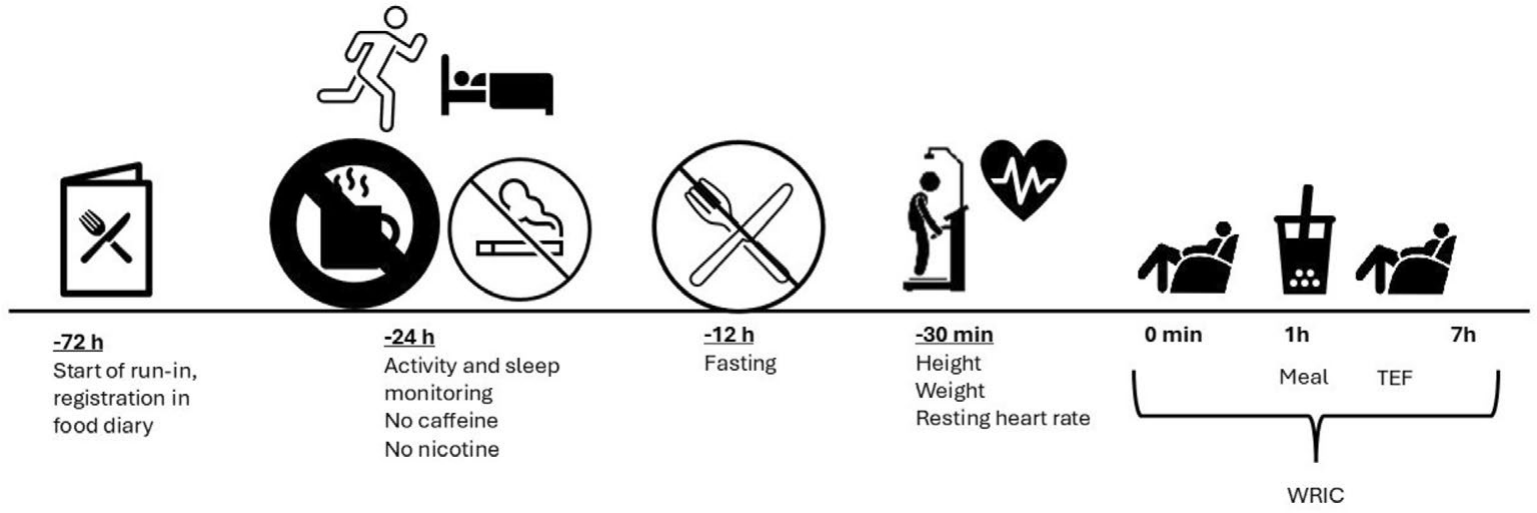
Overview of the study protocol which was repeated twice. TEF, thermic effect of food; WRIC, whole‐room indirect calorimeter.

An overview of the study protocol is shown in Figure 1. Seventy‐two hours before measurement of TEF, the participants were instructed to follow a balanced diet, according to the Norwegian food‐based dietary guidelines (Helsedirektoratet, 2012), and to complete a dietary record. Furthermore, the participants were instructed to consume the same foods 3 days before the second measurement. The recorded foods were coded in the “Food composition database and food and nutrient calculation system (KBS) (database: N4)”, at the Department of Nutrition, University of Oslo. Twenty‐four hours prior to the day of the TEF measurement, the participants were instructed to refrain from caffeine and nicotine and not to engage in vigorous physical activity. They were also instructed to sleep between 6 and 8 h the night before and fast for 12 h before meeting at the study center. To control adherence to physical activity and sleep guidelines, the participants wore an ActiGraph wGT3X‐BT (ActiGraph, Pensacola, FL, USA) on their non‐dominant wrist on the final day before measurement.

Participants arrived at the study center at 07:30 am. Upon arrival, height and weight were measured using a wireless scale (Seca 285, Birmingham, UK). To monitor heart rate, the participants wore a Polar Unite fitness watch (Polar Unite, Polar, Kempele, Finland) on their wrist and a heart rate sensor (Polar H10, Polar, Kempele, Finland) on their chest. These devices recorded both resting, maximum, and average heart rate during the measurement. These measurements were utilized to ensure that the participants were in a resting state during the metabolic test. After a 30‐min rest period, the resting heart rate was recorded, and participants were then escorted to the WRIC, where they were seated in a recliner for the duration of the measurement.

The measurement was started 5 min after the door to the WRIC was closed. The REE was assessed during the first hour. After this initial first hour, the participants were instructed to consume a smoothie within 15 min, where the energy content was equivalent to 25% of their estimated REE, as calculated using the Mifflin‐St Jeor Equation (Mifflin et al., 1990). The macronutrient composition of the smoothie was ~13 E% protein, ~46 E% carbohydrate, ~36 E% fat (Appendix S1). The participants remained seated in the WRIC for the subsequent 6 h, except for using the restroom placed inside the room. They were allowed to perform light activity, such as working on a laptop/reading/watching TV to prevent falling asleep. Participants could drink 1.5 L of water throughout the measurement period.

### 2.6 Body composition

Body composition was measured using dual‐energy X‐ray absorptiometry (DXA) (GE Lunar iDXA, software enCore version 18, GE Healthcare, Madison, WI, USA) between the two measurements in the WRIC. Manufacturer's procedure was followed, including lightweight clothing and removal of all jewelry and other metal artifacts (Alavi et al., 2021; Hangartner et al., 2013; Henriksen et al., 2021).

### 2.7 Statistics

The first and last 10 min of the 7‐h measurements were considered the equilibration period and were removed from analysis, meaning that the whole analysis period was from 10 to 410 min. For the biological validation, REE was measured from 10 to 60 min (Henriksen et al., 2023). To verify fasting adherence prior to measurements, mean RER from the first hour (10–60 min) was calculated.

Statistical analysis was performed in R using RStudio (Purcell et al., 2024; Team RS, 2025) and IBM SPSS Statistics (IBM Corp. (2024), version 30, Armonk, NY, USA). R packages used for data wrangling, plotting and statistical analysis included the *tidyverse* package compilation (Wickham et al., 2019), *data.table* (downloaded from CRAN, version 1.17.0), *lme4* (Bates et al., 2015), *pracma* (downloaded from CRAN, version 2.4.4), *readxl* (downloaded from CRAN, version 1.4.5), and *writexl* (downloaded from CRAN, version 1.5.2). Distribution of continuous variables was checked by visual inspection of histograms and quintile‐quintile plots. All results will be presented as mean ± standard deviation, unless otherwise noted.

Sample size was estimated using the R‐package *ICC.Sample.Size* and the *calculateIccSampleSize* function. We hypothesized an intraclass correlation coefficient (ICC) for TEF of 0.8, with two measurements per subject, alpha of 0.05 and beta of 0.9. This gave a sample size of 10. Other studies of TEF using WRIC have utilized a similar number of subjects (Dörner et al., 2022; Purcell et al., 2024; Ruddick‐Collins et al., 2022).

The VO_2_, VCO_2_, energy expenditure, and RER from the propane combustion tests were calculated as means over the entire duration of the tests (10–410 min), while VO_2_, VCO_2_, and postprandial energy expenditure from the biological measurements were analyzed as area under the curve (AUC) employing the trapezoidal rule from 75 to 410 min. These values were then converted to L/min for VO_2_ and VCO_2_, and kcal/min for postprandial energy expenditure. RER from the biological measurements is presented as mean from 75 to 410 min. To quantify TEF, we first established a mean baseline minute‐by‐minute energy expenditure during the hour prior to meal consumption. This value was then subtracted from the minute‐by‐minute postprandial energy expenditure, setting negative values to zero. The TEF was calculated using AUC over the 335‐min period. To calculate the percent of TEF, the sum of the minute‐by‐minute AUC was divided by the total postprandial energy expenditure of the whole measurement and multiplied by 100.

The participants were instructed to record bathroom visits. In cases where the registered time did not align with the peaks in the data, we performed a visual inspection and applied linear interpolation from approximately 30 s prior to the recorded time until 6 min afterwards.

To assess the accuracy and precision of the WRIC, we calculated the crude relative difference between the repeated measurements and between the measured and expected values for the propane combustion tests alongside the coefficient of variance (CV), expressed as percentage calculated as (standard deviation [SD]/mean) × 100.

For the technical validation, independent *t*‐tests was used to compare measured versus expected VO_2_, VCO_2_, RER, and energy expenditure. The repeated measurements in the biological validation were compared using paired *t*‐tests. Pearson correlation analysis was performed between measured and expected values for VO_2_, VCO_2_, and energy expenditure for the propane combustion tests. The standard deviation for RER was 0, and therefore a coefficient could not be computed. This same analysis was performed for the biological validation but included RER. A similar analysis was performed to determine the relationship of all the above metabolic parameters between the first and second day of the TEF measurements.

We also derived the ICC from linear mixed regression models. The outcome variables were set as the dependent variable, with no independent variable and a random term for subject ID. To express the ICC as a percentage, we multiplied the value by 100. For both Pearson correlation coefficients and the ICCs, a value of >90% was defined as strong or excellent based on previous literature (Allerton et al., 2021). Because physiological RER is constrained to a narrow range, between‐subject variability is inherently limited. As a result, most of the observed variance arises from within‐subject variability, driving down the ICC. In other words, when the total possible spread of true RER values across individuals is small, the ratio of between‐subject variance to total variance becomes low and highly sensitive to even modest within‐subject noise. We therefore recommend that the CV is emphasized when considering the RER values.

For TEF we also calculated the minimal detectable change at 95% confidence interval (MDC_95_) from the standard error of measurement (SEM) as MDC_95_ = SEM × 1.96 × √2, where 1.96 corresponds to the level of confidence adopted (95% in this case) and √2 represents the correction factor for measurement in duplicate.

To visualize the limits of agreement and the presence of outliers in the biological validation, Bland–Altman plots were created. In addition, linear regression models were developed to explore proportional bias, with the difference between day 1 and day 2 for each outcome regressed on the mean value of each parameter for each subject.

## 3 RESULTS

### 3.1 Technical validation

For the five 7‐h propane gas combustion tests, the mean amount propane burned (g) was 66.42 ± 11.02, and the burn rate (g/min) was 0.1581 ± 0.0262. The details from the tests are presented in Table 1. The difference between measured and expected values ranged between −0.33% ± 0.75% for RER and 2.41% ± 2.27% for energy expenditure (Table 1). CV ranged between 0.23% for RER and 1.74% for energy expenditure. There was a near perfect correlation between measured and expected values for VO_2_, VCO_2_, and energy expenditure (*r* = 0.998 for all variables), and there were no significant differences between measured and expected values (*p* > 0.05) (Table 1).

**TABLE 1:** Results from the technical validation (*n* = 5).

	Measured^a^	Expected^a^	∆ (SD)^a^, ^b^	CV (%)^c^	*r* ^d^	*p* ^f^
VO_2_ (L)	160.08 (29.55)	161.01 (26.71)	0.82 (2.35)	1.20	0.998	0.96
VCO_2_ (L)	96.16 (17.29)	96.61 (16.02)	0.63 (1.84)	1.09	0.998	0.97
RER	0.60 (0)	0.60 (0.00)	−0.33 (0.75)	0.23	NA^e^	0.37
EE, kcal	738 (136)	754 (125)	2.41 (2.27)	1.74	0.998	0.85

Abbreviations: CV, coefficient of variation; EE, energy expenditure; RER, respiratory exchange ratio; VCO_2_, ventilation rates for carbon dioxide; VO_2_, ventilation rates for oxygen.

^a^ Values are mean (standard deviation).

^b^ Deltas are measured–expected.

^c^ CVs are standard deviation measured + expected/mean measured + expected ×100.

^d^ Pearson's correlation coefficient between measured and expected values.

^e^ The standard deviation for RER was zero and a coefficient could not be computed.

^f^ *p* Values are derived from independent *t*‐tests comparing measured versus expected values.

### 3.2 Baseline characteristics and protocol adherence

Characteristics of the 10 included participants are presented in Table 2. Of the 10 participants, 6 (60%) were male. The age was 33.5 ± 10.3 years and body mass index 24.1 ± 2.5 kg/m^2^. The participants had a varied diet, with distribution of macronutrients in line with the Norwegian dietary guidelines, and low physical activity according to the ActiGraph measurements the last 24 h prior to measurement in the WRIC. With respect to caffeine intake, one participant consumed 1 dL of Coca‐Cola Zero Sugar in the afternoon, and another consumed 5 dL of coffee in the morning, both on the day preceding measurement day 1. Calculated RER the first hour indicates that the participants adhered to the fasting protocol (Table 3).

**TABLE 2:** Participant characteristics and protocol adherence.

*n* = 10	Mean (SD or %)	Median (min, max)
Males, *n* (%)	6 (60)	
Age, y	33.5 (10.3)	31 (20, 56)
Ethnicity and race		
Caucasian, *n* (%)	9 (90)	
Asian, *n* (%)	1 (10)	
Anthropometric measurements		
Body weight, kg	70.7 (13.2)	72.0 (55.0, 94.8)
Height, cm	170.7 (10.5)	173.2 (154.6, 184.1)
Body mass index, kg/m^2^	24.1 (2.5)	24.2 (20.6, 28.0)
Physical activity and sleep		
Steps, *n*	11,871 (2608)	11533 (7343, 16,746)
Moderate‐to‐vigorous physical activity, min/d	70.3 (48.0)	58.8 (22, 172.5)
Sedentary time, h/d	12.1 (1.76)	12.6 (9.7, 14.8)
Total sleep, h	6.8 (0.80)	6.9 (5.47, 7.93)
Body composition		
Fat mass, kg	16.4 (3.4)	15.5 (9.9, 20.6)
Visceral adipose tissue, g	377.3 (360.6)	271.0 (52, 1081.0)
Subcutaneous adipose tissue, g	863.6 (420.3)	928.5 (115.0, 1514.0)
Fat free mass, kg	54.8 (13.9)	54.7 (36.8, 75.7)
Lean mass, kg	52.0 (13.3)	52.1 (34.6, 71.8)
Dietary intake, *n* = 9^a^		
Energy intake, kcal/d	2043 (549)	2129 (1178, 2860)
Protein, E%	21 (3.1)	21 (15.9, 25)
Carbohydrate, E%	41 (7.7)	44 (28.7, 50.3)
Fat, E%	35 (5.5)	35 (27, 42.7)
Fruit, berries, vegetables, and juice, g/d^b^	663 (223)	626 (346, 971)
Nuts and seeds, g/d	15 (12.6)	12 (0, 31.5)
Whole grains, g/d	84 (61.3)	68 (22.3, 223.6)
Fish (total), g/d	81 (61.3)	57 (0, 179.8)
Meat (total red and white meat), g/d	110 (115.1)	61 (0, 360.1)
Dairy products, g/d^c^	398 (152.8)	414 (174.4, 640.3)
Potatoes, g/d	45 (67.2)	11 (0, 205.1)
Legumes, g/d	5 (9.0)	0 (0, 21)
Sugary foods, g/d^d^	17 (29.5)	4 (0, 88.7)
Margarine, butter, oils, g/d	17 (9.6)	16 (3.5, 32.1)

^a^ One participant did not complete the food diary after end of study.

^b^ Include max one glass (200 grams) of juice as one portion of fruit (100 g), not jam.

^c^ Includes milk, yoghurt, cheese, and other dairy products.

^d^ Includes cakes, dessert, ice cream, chocolate, and candy.

**TABLE 3:** Accuracy and reliability of repeated measurements of energy expenditure and physiological parameters (*n* = 10).

	Day 1	Day 2	∆ (SD)^a^	CV (%)^b^	ICC^c^	*r* ^d^	*p* ^e^
	Mean (SD)	Mean (SD)					
Resting energy expenditure, kcal/d	2152 (396)	2029 (416)	−5.93 (5.25)	4.48	92	96	0.007
Resting energy expenditure, kcal/min	1.49 (0.27)	1.41 (0.29)	−5.93 (5.25)	4.48	92	96	0.007
Mifflin‐St Jeor, kcal/d	1545 (286)	1551 (284)	0.46 (0.83)	0.51	100	100	0.13
Resting heart rate, bpm	58 (8.1)	55 (8.5)	−4.08 (12.6)	5.54	48	49	0.32
Average heart rate, bpm	64 (7.2)	61 (6.4)	−4.84 (4.90)	3.82	79	88	0.017
VO_2_, L/min	0.34 (0.06)	0.34 (0.07)	−2.49 (5.42)	3.42	96	97	0.24
VCO_2_, L/min	0.27 (0.05)	0.27 (0.05)	−0.19 (3.30)	1.97	98	98	0.89
Fasting RER	0.76 (0.04)	0.79 (0.04)	4.72 (6.11)	3.25	17	36	0.037
Postprandial RER	0.78 (0.03)	0.80 (0.03)	2.85 (4.95)	2.58	18	29	0.10
Postprandial energy expenditure, kcal/min	1.65 (0.28)	1.62 (0.32)	−2.09 (4.82)	3.07	96	97	0.28
TEF, kcal^f^	56.75 (23.01)	74.71 (26.91)	44.11 (63.89)	27.48	24	36	0.08
TEF, %	10.28 (3.92)	13.68 (3.97)	45.94 (60.49)	28.15	22	41	0.033

Abbreviations: bpm, beats per minute; CV, coefficient of variation; ICC, intraclass correlation coefficient; RER, respiratory exchange ratio; SD, standard deviation; TEF, thermic effect of food; VCO_2_, ventilation rates for carbon dioxide; VO_2_, ventilation rates for oxygen.

^a^ Deltas, ∆, are expressed as percentages, ((day 2−day 1)/day 1) × 100.

^b^ CVs are (standard deviation between day 1 and day 2/mean between day 1 and day 2) × 100.

^c^ ICCs are derived from linear mixed model regression with the measured parameter as the outcome and a random term for subject ID.

^d^ Pearson's correlation coefficient between measured parameters at day 1 and day 2.

^e^ *p* Values are derived from paired *t*‐tests comparing values between day 1 and day 2.

^f^ kcal is derived from the iAUC for the postprandial measurement period.

### 3.3 Biological reliability

The participants had a measured REE of 1.49 ± 0.27 kcal/min on the first measurement day, and 1.41 ± 0.29 kcal/min on the second measurement day, which translates to 2152 and 2029 kcal/d, respectively. The Mifflin‐St Jeor equation calculated 1545 ± 286 kcal/d and 1551 ± 284 kcal/d on day 1 and day 2, respectively (Table 3). There was a statistically significant elevated average heart rate during the measurements compared to the resting heart rate measured before entering the WRIC. The mean differences in resting heart rate prior to the measurement and average heart rate during the measurement were 5.8 bpm on day 1 (*p* = 0.008) and 5.4 bpm on day 2 (*p* = 0.036).

**FIGURE 2:**
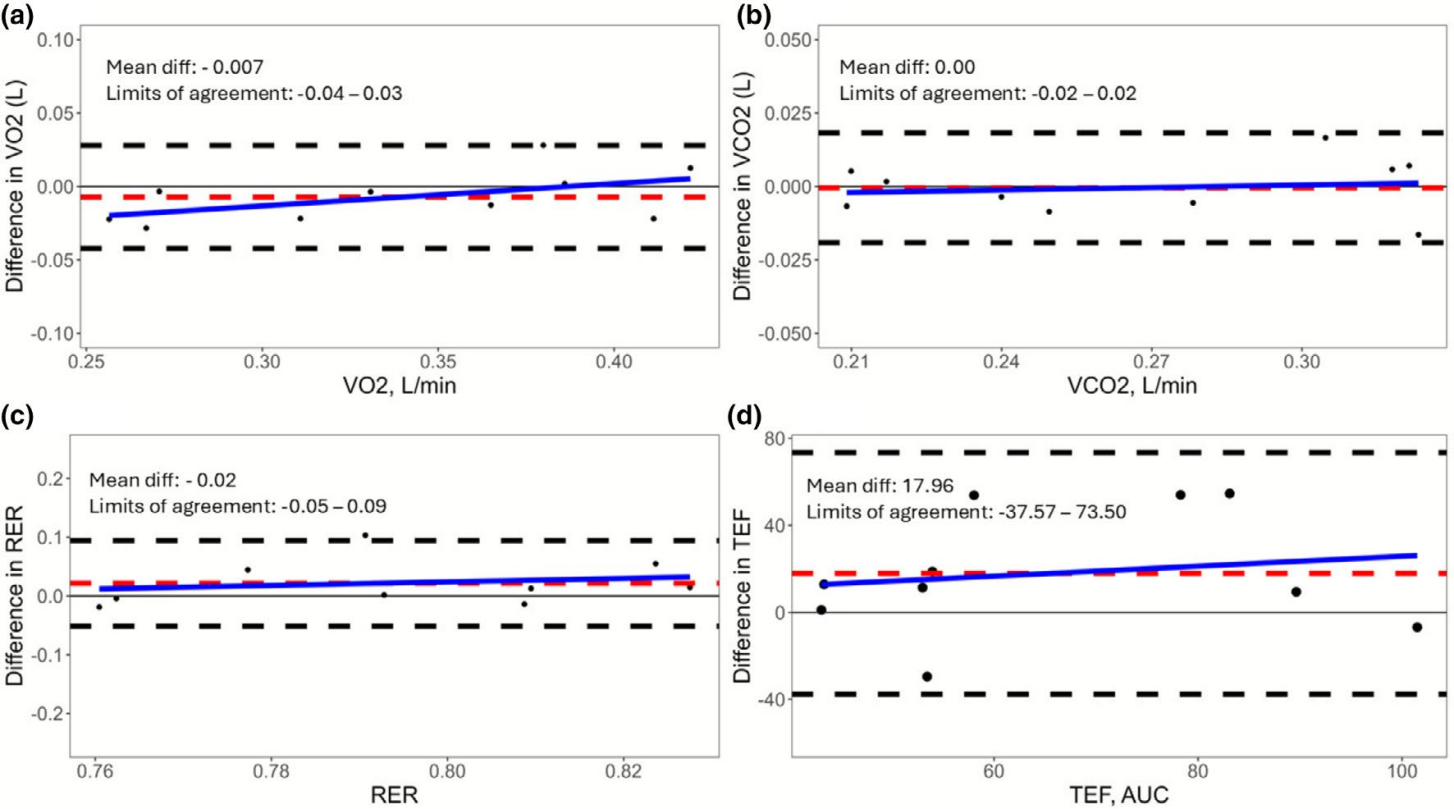
Bland–Altman plots with zero (gray solid line), mean difference (red dashed line), limits of agreement (black dashed lines), and regression line for proportional bias (blue solid line). (a) VO_2_, (b) VCO_2_, (c) RER, and (d) TEF. AUC, area under the curve; RER, respiratory exchange ratio; TEF, thermic effect of food; VCO_2_, ventilation rates for carbon dioxide; VO_2_, ventilation rates for oxygen.

**FIGURE 3:**
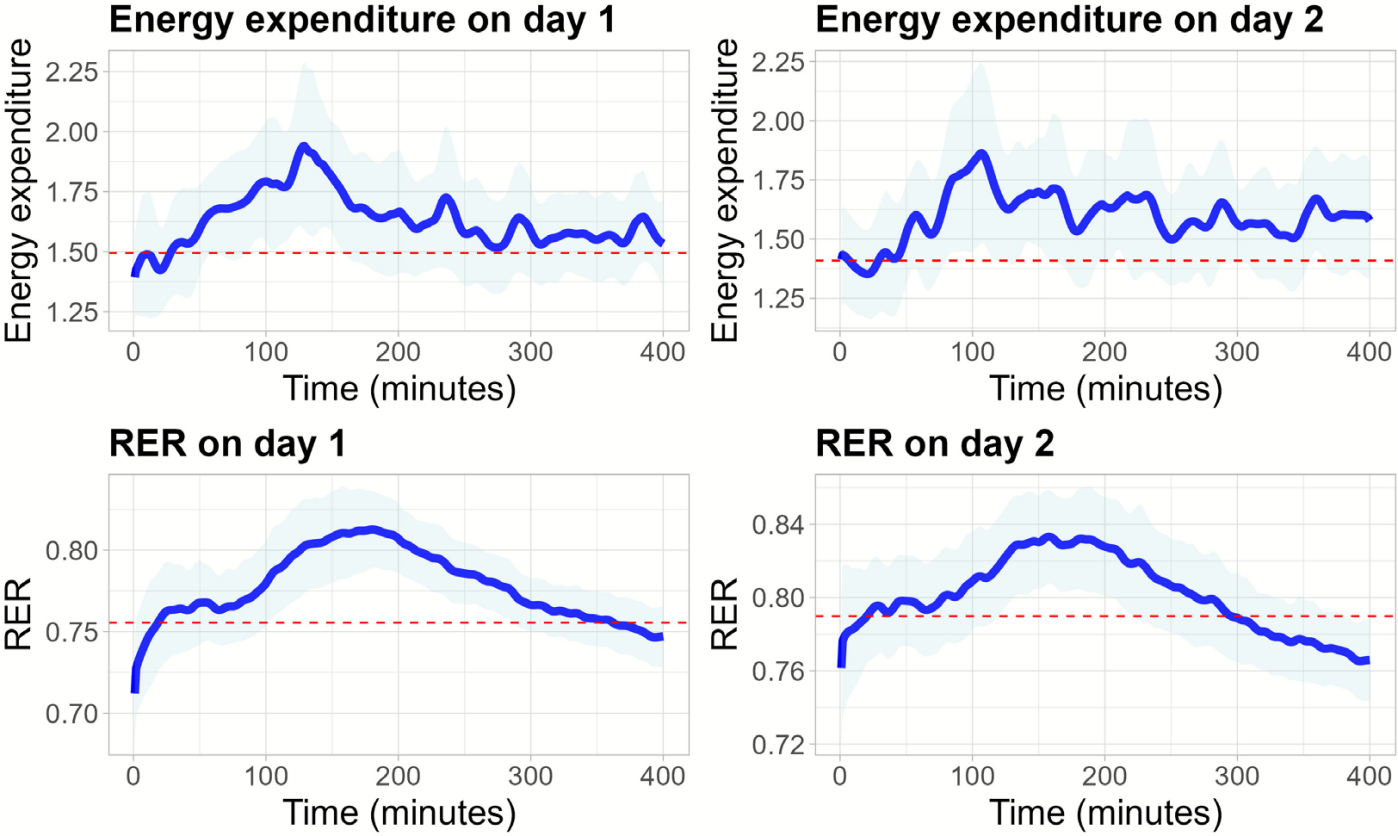
Time series plots for measured energy expenditure and RER at measuring day 1 and day 2. Blue solid line represents the minute‐by‐minute energy expenditure/RER, red dashed line represent the mean baseline value the first hour of measurements, and the light blue outline is the 95% confidence interval. RER, respiratory exchange ratio.

The details of the biological reliability are presented in Table 3. Notably, there was a slightly lower REE at measurement day 2 versus day 1 (*p* = 0.007), but both the CV (4.48%) and ICC (92%) were acceptable. No significant difference between measurement days were observed for postprandial energy expenditure, which demonstrated excellent reliability (ICC 96%). Postprandial VO_2_ and VCO_2_ both showed excellent reliability (ICC 96% and 98%, respectively). In contrast, both fasting and postprandial RER and TEF showed poor reliability over the measurement period (ICC 17%, 18%, and 24%, respectively). There was no evidence of proportional bias, as visualized in Bland–Altman plots (Figure 2). The observed MDC_95_ and MDC% for TEF were 24.93% and 43.76%, respectively. To visualize the variation over time for energy expenditure and RER, time series plots are presented in Figure 3. For the values from the whole period (10–410 min), see Table S1.

## 4 DISCUSSION

This study aimed to evaluate the 7209 L WRIC at the Department of Nutrition, University of Oslo, Norway, for the measurement of VO_2_, VCO_2_, RER, postprandial energy expenditure, and TEF over a 7‐h period utilizing propane combustion and replicate measurements in human subjects. The technical validation showed excellent correlation between measured and expected values for VO_2_, VCO_2_, and energy expenditure, and our results are in line with previous studies (Henriksen et al., 2023; Rising et al., 2017).

For the biological validation, VO_2_, VCO_2_, and postprandial energy expenditure showed good reliability; however, RER and TEF showed lower reliability. Mean TEF was calculated to be 10.3% and 13.7% on measurement day 1 and 2, respectively, and ranged between 4.7% and 19.6%, which is similar to what has been reported previously (Calcagno et al., 2019; Du et al., 2014; Levine, 2005; Ruddick‐Collins et al., 2022). Some of the differences between measured TEF on day 1 and 2 may be attributed to the lower fasting REE on day 2, as the measured postprandial energy expenditure showed good reliability (ICC 96%). The difference in REE between the two measurement days may have contributed to the high variability of the TEF measurements. This difference in REE was not expected but may be a result of shorter measurement periods. Recording REE over a longer period would likely have provided a more stable baseline from which to calculate TEF. Related to this, our short‐term REE measurement is different to prior studies that measured TEF in larger chambers with information about energy expenditure at sleep. Another possible reason for the difference could be that postprandial energy expenditure more frequently dips below the measured baseline during the postprandial measurement. However, our results are consistent with previous studies (Allerton et al., 2021; Dörner et al., 2022; Dulloo et al., 2012; Piaggi et al., 2013; Piers et al., 1995; Ravussin et al., 1986) and further underscore that biological factors are likely to play a significant role in measuring TEF. When TEF is higher, REE might be lower and vice versa, keeping the total energy expenditure relatively constant between measurements, as we see in the excellent ICC for postprandial energy expenditure (Piers et al., 1992).

The MDC% for TEF is similar to findings in a previous study (Dörner et al., 2022), and reflects the inherent variability in TEF measurements. Our results indicate that a substantial change of approximately 44% is required to detect a significant effect on TEF. This emphasizes the challenges associated with identifying meaningful physiological changes in TEF and suggests that interventions aimed to influence TEF must give substantial changes to yield detectable effects. This finding can be particularly relevant for designing future studies and interpreting the effects of dietary interventions on TEF.

The low reliability of RER has also been observed in a previous study using the novel WRIC facility at the University of Oslo (Henriksen et al., 2023). Although the reason for this low reliability of RER is unclear, it is suggested that random errors in VO_2_ and VCO_2_ measurements accumulate, resulting in inaccurate RER measurements (Henriksen et al., 2023; Miles‐Chan et al., 2015) (see also method section). Even with small errors, the ratio can amplify these over time, especially if the values are close together or vary in parallel. The fasting RER values observed in this study were similar to what is reported in Henriksen et al. (2023) and Rising et al. (2015), suggesting that the participants were likely in a fasting state. A RER value under 0.8 is typically indicative of fasting; however, some participants had fasting RER as high as 0.86. It is important to note that fasting RER can be affected by dietary intake the day before measurement (Hill et al., 1991; Miles‐Chan et al., 2015), as digestion, absorption, and metabolism may still be occurring by the time of the REE measurement in some subjects. Additionally, physical activity performed the day before could also impact fasting RER, as it may alter substrate utilization and metabolic state in the hours leading up to measurement. However, the participants were instructed to avoid vigorous physical activity the day prior, and ActiGraph data indicates that they adhered to this guideline, and there were no differences in recorded activity the day before the two measurement days. Furthermore, participants were instructed to refrain from caffeine the last 24 h before measurement, and for habitual caffeine consumers, withdrawal effects may impact measured energy expenditure and RER (Sajadi‐Ernazarova & Hamilton, 2025).

Measurements of TEF have often been conducted within larger WRICs as part of 24‐h measurements (Allerton et al., 2021; Dörner et al., 2022; Dulloo et al., 2012; Piaggi et al., 2013; Ravussin et al., 1986; Tataranni et al., 1995; Usui et al., 2015). Such large WRICs can diminish the detectability of subtle changes in energy expenditure after a meal due to the size of the room. By using a smaller WRIC, we hypothesized that this would mitigate the errors due to dilution effects and shorten response times, subsequently reducing the need for extensive correction related to room volume. This methodology could potentially enhance precision; however, we did not observe these results, as shown by the high CV and lower ICC for TEF. This shows that even with excellent reproducibility for postprandial energy expenditure, VO_2_, and VCO_2_, we are not able to capture TEF.

Moreover, studies often provided meals with shorter intervals than 6 h, which complicates the accurate registration of the entire TEF duration and may result in overlapping responses from consecutive meals (Compher et al., 2006; Piaggi et al., 2013; Ruddick‐Collins et al., 2022). Our study only provided one meal after an overnight fast, and measures for 6 h postprandially, which increases the chances of measuring the entire period of TEF. However, in Figure 3, we observed that the energy expenditure did not return to baseline REE after the 6 h postprandially, meaning that even with the long period, we might not have captured the whole period of TEF for all participants.

Studies utilizing ventilated hoods or face masks to measure TEF (Jones et al., 2004; Piers et al., 1995), reduce the participants' comfort and increase stress during longer measurements. These methods often require subjects to remain still and rely on achieving a steady‐state condition, which can introduce variability in the data. In contrast, WRICs minimize discomfort and stress for participants during extended measurements, allow for continuous monitoring, and offer improved measurement accuracy over time. Studies have shown that WRICs provide more stable and precise estimates of energy expenditure, including TEF, compared to ventilated hood and face mask systems, particularly during long‐duration assessments (Rising et al., 2015; Van Soom et al., 2023).

To calculate the amount of smoothie the participants should drink (25% of the estimated REE), we used the Mifflin‐St Jeor Equation (Mifflin et al., 1990) to estimate REE. After calculating the REE during the first hour within the WRIC, we observed a significant difference between the estimates provided by the Mifflin‐St Jeor equation and the measurements obtained from the WRIC (data not shown). Specifically, the Mifflin‐St Jeor equation underestimated the REE compared to the measurements from the WRIC. Consequently, this discrepancy may have led to participants receiving less than the intended 25% of their actual REE. To address this in future studies, we could consider inviting participants for an additional visit prior to the experimental visits to measure REE and ensure they receive 25% of REE measured by the WRIC.

We observed lower measured REE, resting heart rate, and average heart rate on the second measurement day compared to the first. This may be attributed to participants becoming more familiar with the procedures and the lab environment.

A limitation in our validation of the WRIC for TEF measurement is that the participants only recorded their intake prior to the first TEF measurement to reduce the participant burden; hence we do not know whether they followed protocol or not before day 2. However, the participants were instructed to follow the Norwegian dietary guidelines (Helsedirektoratet, 2012) and to record the food intake 3 days prior to day 1, repeating this for the 3 days leading up to day 2. While they only recorded their intake prior to the first TEF measurement, the resulting data provide valuable insight into their compliance. Although we do not have information on their adherence to the diet before day 2, the findings in our biological validation are promising. Implementing greater control by requiring dietary records before day 2 or providing standardized meals could have further enhanced the results and strengthened our conclusions.

We did not record movement inside the room during measurement. Although the design facilitated little movement and the participants were instructed to remain resting the whole period, we cannot rule out the potential influence of smaller movements on our measurements. We did find a significant difference in the measured resting and average heart rate at both measuring days; however, the heart rate does increase after the consumption of a meal, and the increase can last for a couple of hours (de Mey et al., 1989). The mean difference between the resting and average heart rate was below 6 bpm. In contrast, some studies measure physical activity and apply energy expenditure/activity regression‐based methods with intercept to estimate TEF (Dörner et al., 2022; Usui et al., 2015), and such methodological differences should be considered when comparing results across studies.

The room's effective air volume has not been determined using washout tests; however, the method used is accepted by the RICORS 1.0 guidelines (Chen et al., 2020). Temperature and humidity were not measured during the measurements; however, the heating, ventilation, and air conditioning system was set to keep a steady temperature of 22 degrees Celsius and dries the air. The 2 measuring days for the participants were also close in time, and we would not think the weather or humidity outside would impact our results.

## 5 CONCLUSION

In summary, our study has illustrated that the WRIC exhibits excellent validity for VO_2_, VCO_2_, RER, and energy expenditure. It also exhibits excellent reliability in assessing VO_2_, VCO_2_, and postprandial energy expenditure. However, the reliability for RER and TEF was found to be lower, even with a smaller room volume. These results hold significant implications for future research and clinical practices, particularly when TEF is the primary outcome.

## AUTHOR CONTRIBUTIONS

TO conceived the idea of the study; MMM, SMU, CH, and TO designed the study; MMM, EMSA, and TO collected data; RR customized the macro files used to estimate metabolic rates; MMM drafted the paper. All authors read, revised, and approved the final version of the paper.

## FUNDING INFORMATION

This project received support from The Throne Holst Foundation for Nutrition Research and the University of Oslo (UiO).

## CONFLICT OF INTEREST STATEMENT

The authors declare no conflict of interest.

## ETHICS STATEMENT

All participants sigend informed consent before the study and all procedures were approved by the Data Protection Services (SIKT) (Reference number: 164609).

## Supporting information


Appendix S1.

## Data Availability

The data that support the findings of this study are sensitive and not openly available. They can be accessed from the corresponding author upon reasonable request. Data are stored in the Service for sensitive data (TSD) at the University of Oslo.
