# Supplementary material for: Validity and reproducibility of a whole‐room indirect calorimeter for measurement of the thermic effect of food
Source: Physiol Rep. 2026 Feb 19;14(4):e70740. doi: 10.14814/phy2.70740 (PMC12917861; doi:10.14814/phy2.70740)
Supplement: Supplementary file 1 — Appendix S1. [file PHY2-14-e70740-s001.docx]

# Supporting Information

**Supporting Information 1: Recipe smoothie, 1 batch = 762g**

- 50g gluten free oats
- 120g banana
- 17g canola oil
- 150g fresh blueberries
- 125g Yoplait 0% fat, blueberry
- 300g milk 3.5% fat (Tine)

**Supporting Table 1:** Accuracy and reliability of repeated measurements (n=10), 10-410 minutes

|  | **Day 1**  **Mean (SD)** | | **Day 2**  **Mean (SD)** | | **∆ (SD)^a^** | | **CV (%)^b^** | **ICC (%)^c^** | ***r*^d^** | **p^e^** |
| --- | --- | --- | --- | --- | --- | --- | --- | --- | --- | --- |
| **VO_2_, L/min** | 0.34 | (0.06) | 0.33 | (0.07) | -0.08 | (5.15) | 3.50 | 96 | 97 | 0.12 |
| **VCO_2_, L/min** | 0.26 | (0.05) | 0.26 | (0.05) | -0.68 | (3.07) | 1.90 | 98 | 98 | 0.54 |
| **RER** | 0.78 | (0.03) | 0.80 | (0.03) | 3.11 | (5.03) | 2.61 | 17 | 30 | 0.08 |
| **Energy expenditure, kcal/min** | 1.63 | (0.27) | 1.59 | (0.31) | -2.66 | (4.55) | 3.14 | 97 | 98 | 0.13 |
| **Energy expenditure, AUC** | 652 | (110) | 637 | (124) | -2.66 | (4.55) | 3.14 | 97 | 98 | 0.13 |

Abbreviations: CV, coefficient of variation; ICC, intraclass correlation coefficient; RER, respiratory exchange ratio; SD, Standard deviation; VCO_2_, ventilation rates for carbon dioxide; VO_2_, ventilation rates for oxygen.

^a^Deltas, ∆, are expressed as percentages, ((day 2— day 1)/day 1) *100.

^b^CVs are (standard deviation between day 1 and day 2/mean between day 1 and day 2) × 100

^c^ICCs are derived from linear mixed model regression with the measured parameter as the outcome and a random term for subject ID.

^d^Pearson's correlation coefficient between measured and parameters at day 1 and day 2.

^e^p-values are derived from paired t-tests comparing values between day 1 and day 2.
